# Effect of Annual Influenza Vaccination on the Risk of Lung Cancer Among Patients With Hypertension: A Population-Based Cohort Study in Taiwan

**DOI:** 10.3389/ijph.2023.1605370

**Published:** 2023-10-02

**Authors:** Hung-Chang Jong, Jing-Quan Zheng, Cai-Mei Zheng, Cheng-Hsin Lin, Chun-Chih Chiu, Min-Huei Hsu, Yu-Ann Fang, Wen-Rui Hao, Chun-Chao Chen, Tsung Yeh Yang, Kang-Yun Lee, Ju-Chi Liu

**Affiliations:** ^1^ Division of Cardiology, Department of Internal Medicine, Shuang Ho Hospital, Taipei Medical University, New Taipei City, Taiwan; ^2^ Graduate Institute of Clinical Medicine, College of Medicine, Taipei Medical University, Taipei, Taiwan; ^3^ Division of Pulmonary Medicine, Department of Internal Medicine, Shuang Ho Hospital, Taipei Medical University, New Taipei City, Taiwan; ^4^ Division of Pulmonary Medicine, Department of Internal Medicine, School of Medicine, College of Medicine, Taipei Medical University, Taipei, Taiwan; ^5^ Department of Internal Medicine, School of Medicine, College of Medicine, Taipei Medical University, Taipei, Taiwan; ^6^ Division of Nephrology, Department of Internal Medicine, Shuang Ho Hospital, Taipei Medical University, New Taipei City, Taiwan; ^7^ Taipei Medical University Research Center of Urology and Kidney, Taipei Medical University, Taipei, Taiwan; ^8^ Taipei Heart Institute, Taipei Medical University, Taipei, Taiwan; ^9^ Division of Cardiovascular Surgery, Department of Surgery, Shuang Ho Hospital, Taipei Medical University, New Taipei City, Taiwan; ^10^ Division of Cardiovascular Surgery, Department of Surgery, School of Medicine, College of Medicine, Taipei Medical University, Taipei, Taiwan; ^11^ Division of Cardiology, Department of Internal Medicine, School of Medicine, College of Medicine, Taipei Medical University, Taipei, Taiwan; ^12^ Graduate Institute of Data Science, College of Management, Taipei Medical University, Taipei, Taiwan; ^13^ Department of Neurosurgery, Shuang Ho Hospital, Taipei Medical University, Taipei, Taiwan; ^14^ Graduate Institute of Medical Sciences, College of Medicine, Taipei Medical University, Taipei, Taiwan

**Keywords:** lung cancer, prevention, hypertension, influenza vaccination, malignancy

## Abstract

**Objectives:** Lung cancer is a main contributor to all newly diagnosed cancers worldwide. The chemoprotective effect of the influenza vaccine among patients with hypertension remains unclear.

**Methods:** A total of 37,022 patients with hypertension were retrospectively enrolled from the Taiwan National Health Insurance Research Database. These patients were further divided into a vaccinated group (*n* = 15,697) and an unvaccinated group (*n* = 21,325).

**Results:** After adjusting for sex, age, comorbidities, medications, level of urbanization and monthly income, vaccinated patients had a significantly lower risk of lung cancer occurrence than unvaccinated patients (adjusted hazard ratio [aHR]: 0.56, 95% confidence interval [CI]: 0.47–0.67). A potential protective effect was observed for both sexes and in the elderly age group. With a greater total number of vaccinations, a potentially greater protective effect was observed (aHR: 0.75, 95% CI 0.60–0.95; aHR: 0.66, 95% CI: 0.53–0.82; aHR: 0.26, 95% CI: 0.19–0.36, after receiving 1, 2–3 and ≥4 vaccinations, respectively).

**Conclusion:** Influenza vaccination was associated with a lower risk of lung cancer among patients with hypertension. The potentially chemoprotective effect appeared to be dose dependent.

## Introduction

Hypertension, a known risk factor for cardiovascular diseases, chronic kidney disease, cognitive impairment and dementia [1], is related to chronic morbidity and mortality [2]. The prevalence of hypertension continues to increase worldwide and is associated with aging and sedentary lifestyles [3, 4]. The lifetime risk of hypertension is nearly 90% [5], and the number of patients with hypertension is projected to increase from approximately 972 million (26.4%) in 2000 to approximately 1.56 billion (29.2%) in 2025, a 60% increase within 25 years [6, 7]. A prospective study verified that hypertension contributes to a larger mortality burden than any other known risk factor [8]. Recently, many clinical studies have reported hypertension as a significant risk factor for various cancers, including breast cancer [9], lung cancer [10], kidney cancer, head and neck cancer, and esophageal cancer [11, 12]. Studies have also revealed a significant positive relationship between the duration of hypertension and the risk of cancer [13–15]**.** The pathologic mechanism underlying chronic hypertension in relation to cancer risk remains unclear, although studies have proposed that chronic hypertension is related to aberrant angiogenesis, which significantly increases the risk of tumorigenesis [12, 15–17]. Hypertension-associated chronic inflammation, oxidative stress and hypertension-inducible mediators, such as vascular endothelial growth factors (VEGFs), hypoxic inducible factors (HIFs), the renin-angiotensin-aldosterone system (RAAS), and insulin-like growth factors (IGFs), might also play roles in tumor development [12]. Common antihypertensive medications are considered to increase the risk of cancer development, and studies have verified that hypertension-related chronic illnesses, including diabetes mellitus (DM), dyslipidemia, and chronic lung diseases, increase the risk of some cancers.

Patients with hypertension have a significantly higher risk of acute viral respiratory infection and serious cardiovascular complications after infection [18]. Although the exact mechanism is unclear, the immune response to the influenza virus may lead to an inflammatory reaction against body tissues and blood vessels [19]. Clinical evidence also indicates that influenza vaccination significantly reduces mortality from cardiovascular and cerebrovascular events [20–22]. Thus, the Taiwanese government has recommended an annual government-funded influenza vaccination for high-risk individuals since 1998 [23]. In our previous studies, we observed that influenza vaccination could protect patients with chronic lung disease [24] and diabetes mellitus [25] from the risk of lung cancer. Whether influenza vaccination reduces the risk of lung cancer among patients with hypertension has yet to be determined. Thus, in this study, we evaluated whether influenza vaccination could reduce the risk of lung cancer among patients with hypertension using a Taiwanese population dataset.

## Methods

Taiwan’s National Health Insurance (NHI) program was established in 1995 and currently provides comprehensive health insurance for more than 98% (>23 million people) of Taiwan’s population. This study analyzed data from between 2000 and 2012 obtained from the NHI Research Database (NHIRD). No significant differences were revealed in terms of age, sex, or healthcare costs between all the participants in the study group and patients in the NHI program. Data from the NHIRD that could be used to identify patients or care providers, including medical institutions and doctors, are encrypted before being sent to the National Health Research Institutes for inclusion in the database, and records are further anonymized before being released to researchers. Theoretically, data cannot be used to identify individuals. All researchers using the NHIRD and its subsets must sign a written agreement stating that they have no intention of obtaining information that might infringe on the privacy of patients or care providers. This study was approved by the Joint Institutional Review Board of Taipei Medical University (approval no. N201804043, on 26 April 2018).

In Taiwan, the influenza vaccination has been free of charge and recommended for high-risk adults aged ≥50 years (i.e., those with type 2 diabetes, chronic liver infection or cirrhosis, cardiovascular diseases, or chronic pulmonary diseases) since 1998 and for all adults aged >65 years since 2001. The vaccination status was identified using the International Classification of Diseases, Ninth Revision, Clinical Modification (ICD-9-CM) code V048 or according to the use of vaccines (confirmed through NHI Drug Codes of influenza vaccines) [26].

The potential confounders included diabetes, dyslipidemia, medications (antihypertensive agents included calcium channel blockers, beta-blocking agents, renin-angiotensin-aldosterone system inhibitors, aspirin, statins, and metformin) with different durations of usage (cumulative prescription <28 days, 28–365 days, and >365 days), sociodemographic characteristics [age, sex, urbanization level [27], and monthly income for each individual before entering this study. In addition, each individual’s Charlson Comorbidity Index (CCI) was assessed by age and comorbidities such as myocardial infarction, congestive heart failure, peripheral vascular disease, cerebrovascular disease, dementia, chronic pulmonary disease, rheumatologic disease, peptic ulcer disease, liver disease, diabetes, hemiplegia or paraplegia, renal disease, malignancy, leukemia, lymphoma and acquired immunodeficiency syndrome.

**FIGURE 1:**
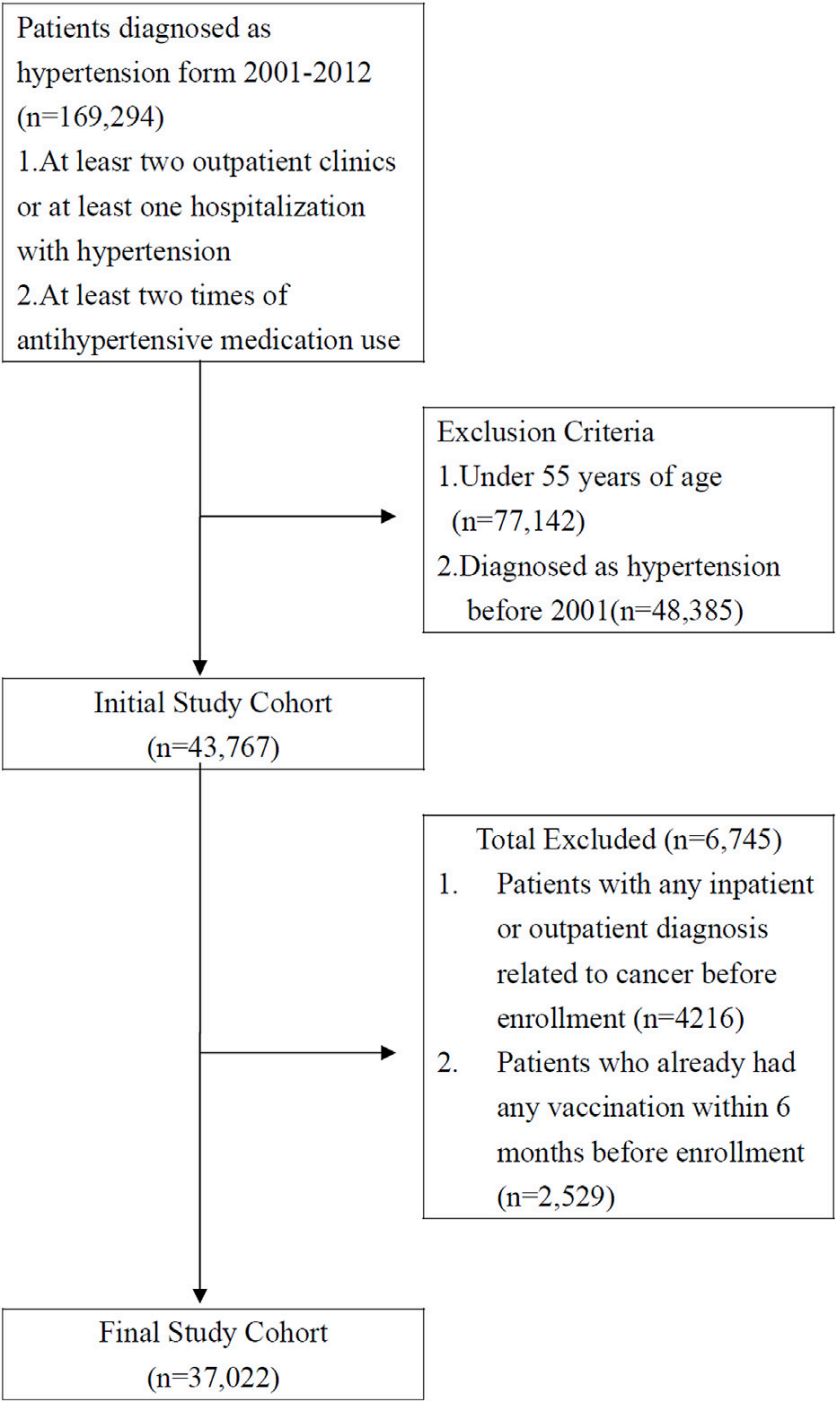
Data selection process (Taiwan, 2000–2012).

The study cohort comprised patients who had received a diagnosis of hypertension (ICD-9-CM codes 401-405) at medical institutions in Taiwan over a 12 years period (*n* = 206,077) between 1 January 2001, and 31 December 2012. Patients who had received a diagnosis of hypertension at more than two outpatient clinics or more than one hospitalization and who had recently taken two or more antihypertensive medications (*n* = 169,294) were recruited for this study. The exclusion criteria were as follows: 1. patients under 55 years old (*n* = 77, 142); 2. To minimize the potential confounding factors associated with previous hypertension status and treatment status of hypertension, patients who had received a diagnosis of hypertension before 2001 (*n* = 48,385) were excluded. 3. To eliminate the confounding effect from other types of cancer, patients who had received a diagnosis related to cancer in any inpatient or outpatient setting (*n* = 4,216) were excluded. 4. Patients who had already received any vaccination within 6 months before the enrollment date (*n* = 2,529) were excluded. Our final study cohort comprised 37,022 patients with hypertension who were older than 55 years in Taiwan. Of these, 15,697 had received an influenza vaccination, and 21,325 had not (Figure 1).

### The Primary Endpoint

**FIGURE 2:**
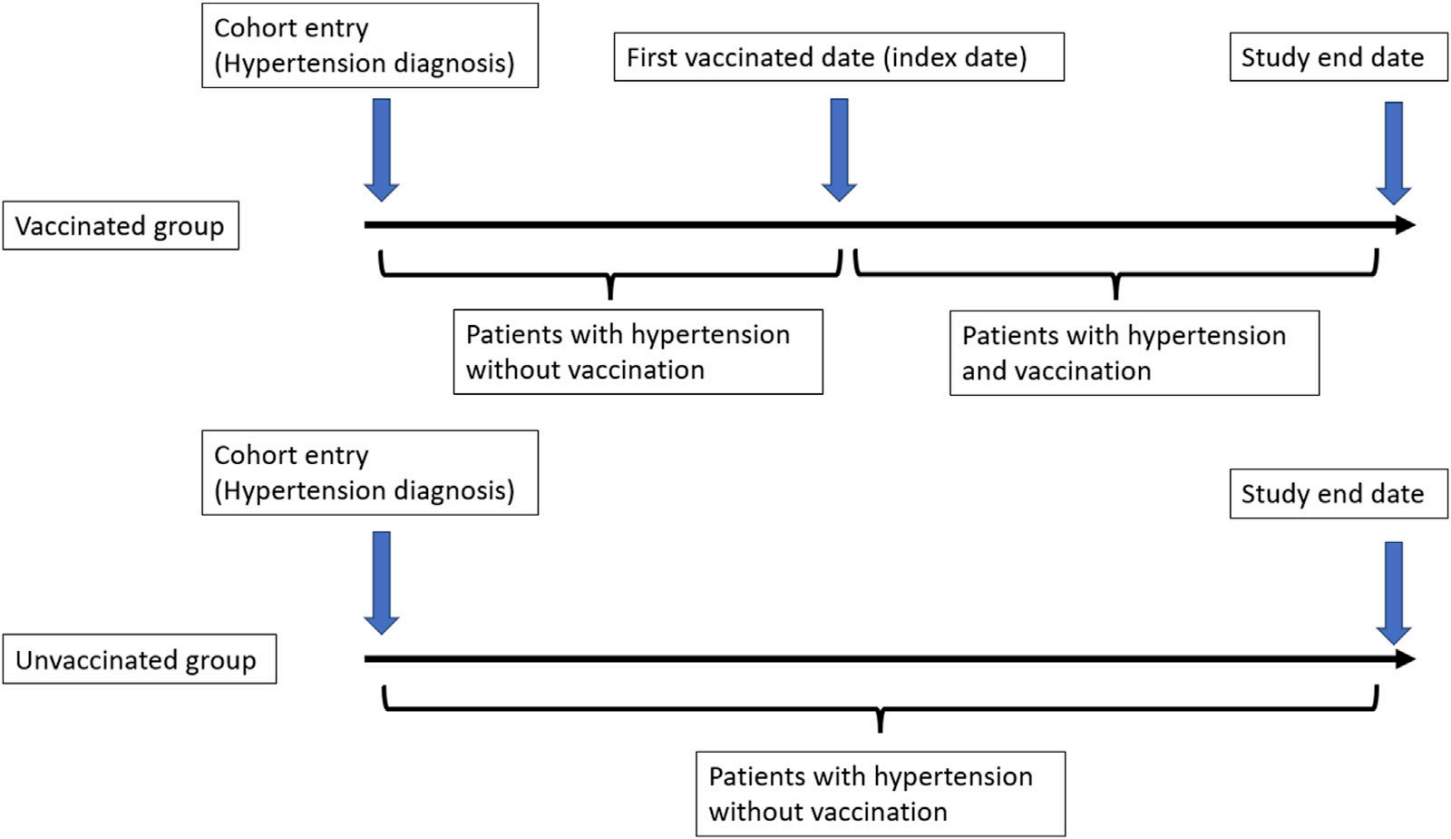
Definition of cohort entry date and index date (Taiwan, 2000–2012).

All subjects were followed up from the date of cohort entry, and all subjects were classified as unvaccinated until their vaccination date, after which their vaccination status changed to vaccinated (Figure 2). The primary endpoint of our study was the incidence of lung cancer (ICD-9-CM code 162.X) among patients with hypertension. All cohorts were followed up until the date of a diagnosis of lung cancer, death, disenrollment from the NHI, or the end of 2012. A total cumulative number of vaccinations before the primary endpoint was calculated in the vaccinated group.

### Statistical Analysis

The propensity score (PS) method was used to reduce the selection bias in the comparison of the vaccinated group and the non-vaccinated group by accounting for the covariates with a logistic regression model [28]. In addition, a time-varying Cox model was used to calculate the hazard ratios (HRs) to determine the risk of lung cancer between the vaccinated and unvaccinated groups [29]. The aforementioned confounders as well as time-varying vaccination exposure and propensity score were included in the Cox model. A chi-square test was used for categorical variables, and the t-test was used for continuous variables.

To examine the effects of the influenza vaccine among patients of different ages and sexes and with different comorbidities and medication usages, a subgroup analysis was performed. To assess the similarities and differences among the patients with hypertension in terms of receipt of the influenza vaccine and the risk of lung cancer, these data were stratified according to patient age (55–64, 65–74, ≥75 years old), sex, comorbidities (with or without diabetes, with or without dyslipidemia, CCI 0, 1, 2, ≥3) and medication (antihypertensive agents, statins, metformin, aspirin) use of different durations.

The dose-dependent effect of influenza vaccinations on the incidence of lung cancer was also determined through a subgroup analysis, in which patients with hypertension were categorized into four groups according to their vaccination status (unvaccinated and total number of vaccinations: 1, 2 and 3, and ≥4).

All analyses were performed using SPSS 22.0 and SAS 9.4 software, and a two-tailed test result of *p* < 0.05 was considered significant.

## Results

The study cohort comprised 37,022 patients, of whom 15,697 (42.4%) had received an influenza vaccination and 21,325 (57.6%) had not (Table 1). The total follow-up period was 59,492 and 303,937 person-years for the unvaccinated and vaccinated patients, respectively. A significant difference was revealed between the two groups in terms of age, sex, use of antihypertensive medications, comedications, level of urbanization and monthly income (Table 1). The prevalence of certain preexisting medical comorbidities, including the patients’ CCI values, was higher in the vaccinated group. In addition, a higher proportion of the vaccinated group was prescribed antihypertensive medication (including beta-blocking agents, diuretics, calcium channel blockers, RAAS inhibitors, and other types of antihypertensive drugs). An analysis of comorbidity-associated medication use revealed that the vaccinated group had used statins, metformin and aspirin longer than the unvaccinated group.

**TABLE 1:** Characteristics of the sample population (Taiwan, 2000–2012).

	Whole cohort (*n* = 37,022)		Unvaccinated (*n* = 21,325)		Vaccinated (*n* = 15,697)	
	*n*	%	*n*	%	*n*	%
Age, years (Mean ± SD)	66.37 (8.08)		64.04 (8.05)		69.52 (6.99)	
55–64	18,511	50.00	13,907	65.21	4,604	29.33
65–74	12,618	34.08	4,884	22.90	7,734	49.27
≥75	5,893	15.92	2,534	11.88	3,359	21.40
Sex						
Female	18,373	49.63	10,378	48.67	7,995	50.93
Male	18,649	50.37	10,947	51.33	7,702	49.07
CCI^a^						
0	16,843	45.5	9,951	46.66	6,892	43.91
1	9,884	26.7	5,622	26.36	4,262	27.15
2	5,695	15.4	3,213	15.07	2,482	15.81
≥3	4,600	12.4	2,539	11.91	2,061	13.13
Diabetes						
No	29,118	78.65	16,783	78.70	12,335	78.58
Yes	7,904	21.35	4,542	21.30	3,362	21.42
Dyslipidemia						
No	28,895	78.05	16,487	77.31	12,408	79.05
Yes	8,127	21.95	4,838	22.69	3,289	20.95
Antihypertension medications						
Other Class of Antihypertensive Drug	7,154	19.32	3,291	15.43	3,863	24.61
Diuretics	18,450	49.84	9,318	43.70	9,132	58.18
Beta-blocking Agents	19,043	51.44	10,217	47.91	8,826	56.23
Calcium Channel Blockers	25,891	69.93	14,105	66.14	11,786	75.08
Renin-angiotensin-aldosterone system inhibitors	22,178	59.90	11,896	55.78	10,282	65.50
Comedications						
Statins						
<28 days	27,284	73.70	16,085	75.43	11,199	71.34
28–365 days	6,124	16.54	3,472	16.28	2,652	16.89
>365 days	3,614	9.76	1,768	8.29	1,846	11.76
Metformin						
<28 days	29,674	80.15	17,300	81.13	12,374	78.83
28–365 days	2,797	7.55	1,724	8.08	1,073	6.84
>365 days	4,551	12.29	2,301	10.79	2,250	14.33
Aspirin						
<28 days	21,745	58.74	13,765	64.55	7,980	50.84
28–365 days	8,458	22.85	4,511	21.15	3,947	25.14
>365 days	6,819	18.42	3,049	14.30	3,770	24.02
Level of Urbanization						
Urban	25,030	67.61	15,421	72.31	9,609	61.22
Suburban	7,992	21.59	4,146	19.44	3,846	24.50
Rural	4,000	10.80	1,758	8.24	2,242	14.28
Monthly income (NT$)						
0	4,327	11.69	2,150	10.08	2,177	13.87
1–19,200	11,477	31.00	6,078	28.50	5,399	34.40
19,200–25,000	11,832	31.96	6,066	28.45	5,766	36.73
≥25,001	9,386	25.35	7,031	32.97	2,355	15.00

^a^ CCI, Charlson comorbidity index.

NT$ (New Taiwan Dollar).

**FIGURE 3:**
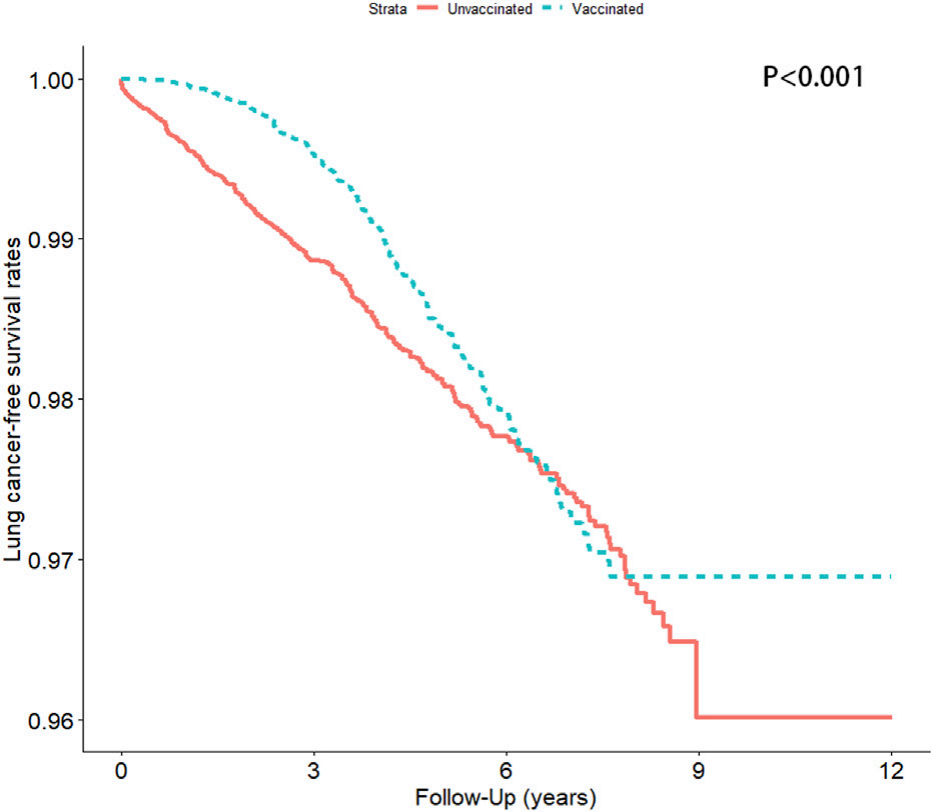
Lung cancer-free survival rate in the vaccinated and unvaccinated groups (Taiwan, 2000–2012).

We analyzed the incidence of lung cancer among patients with hypertension who had and had not received an influenza vaccination (Table 2 and Figure 3). After adjustment for potential confounders, the stratified analysis revealed that the incidence of lung cancer in the vaccinated group was significantly lower than that in the unvaccinated group [the adjusted hazard ratio (aHR) was 0.65, and the 95% confidence interval (95% CI) was 0.55–0.77], particularly among patients aged ≥65 years, irrespective of sex.

**TABLE 2:** Risk of lung cancer among unvaccinated and vaccinated patients in the study cohort (Taiwan, 2000–2012).

Overall group (*n* = 128,348)	Unvaccinated (Total follow-up 96,626.9 person-years)					Vaccinated (Total follow-up 94,366.2 person-years)				Adjusted HR^a^ (95% CI)
	No. of patients	No. of patients with cancer	Incidence rate (per 10^5^ person-years) (95% CI)			*N*	No. of patients with cancer	Incidence rate (per 10^5^ person-years) (95% CI)		
Whole cohort										
Study cohort	21,325	379	392.2	(352.7	431.7)	15,697	255	270.2	(237.1, 303.4)	0.65 (0.55, 0.77)
Age 55–64^b^										
Study cohort	13,907	172	276.3	(235.0	317.6)	4,604	63	207.3	(156.1, 258.5)	0.85 (0.63, 1.14)
Age 65–74^c^										
Study cohort	4,884	120	512.1	(420.5	603.8)	7,734	125	272.3	(224.5, 320.0)	0.60 (0.47, 0.78)
Age ≥75^d^										
Study cohort	2,534	87	795.1	(628.0	962.2)	3,359	67	370.9	(282.1, 459.7)	0.52 (0.38, 0.72)
Female^e^										
Study cohort	10,378	143	293.5	(245.4	341.6)	7,995	93	190.3	(151.6, 229.0)	0.64 (0.49, 0.85)
Male^f^										
Study cohort	10,947	236	492.7	(429.8	555.6)	7,702	162	356.0	(301.2, 410.9)	0.66 (0.53, 0.81)

CI, confidence interval; HR, hazard ratio.

^a^ Main model was adjusted for age, sex, CCI, diabetes, dyslipidemia, antihypertensives, diuretics, beta-blocking agents, calcium channel blockers, RAAS inhibitors, statins, metformin, aspirin, level of urbanization, and monthly income in the propensity score.

^b^ Total follow-up 62,254.4 person-years for unvaccinated and 30,393.7 for vaccinated individuals.

^c^ Total follow-up 23,430.8 person-years for unvaccinated and 45,908.6 person-years for vaccinated individuals.

^d^ Total follow-up 10,941.7 person-years for unvaccinated and 18,063.9 person-years for vaccinated individuals.

^e^ Total follow-up 48,727.6 person-years for unvaccinated and 48,864.1 for vaccinated individuals.

^f^ Total follow-up 47,899.3 person-years for unvaccinated and 45,502.1 for vaccinated individuals.

An additional subgroup analysis was performed to evaluate the association between influenza vaccination and the risk of lung cancer in different models, with stratification according to the total number of vaccinations (Table 3). Notably, a dose-dependent protective effect was observed in the main model, with a significant protective effect identified in those who had received ≥4 vaccinations (aHRs [95% CI] = 0.54 [0.40, 0.74]).

**TABLE 3:** Subgroup analysis of adjusted HRs of vaccination in risk reduction of lung cancer in all seasons (Taiwan, 2000–2012).

	Unvaccinated		Vaccinated						P for trend
			1		2–3		≥4		
	*n*	Adjusted HR (95% CI)	*n*	Adjusted HR (95% CI)	*n*	Adjusted HR (95% CI)	*n*	Adjusted HR (95% CI)	
Main model^a^	21,325	1.00	5,106	0.64 (0.51, 0.80)***	5,778	0.72 (0.58, 0.90)**	4,813	0.54 (0.40, 0.74)***	<0.001
Subgroup effects									
Age, years									
55–64	13,907	1.00	1,886	0.72 (0.48, 1.09)	1,696	0.89 (0.58, 1.37)	1,022	1.20 (0.66, 2.17)	0.722
65–74	4,884	1.00	2,113	0.68 (0.48, 0.96)*	2,787	0.61 (0.44, 0.85)**	2,834	0.49 (0.32, 0.74)***	<0.001
≥75	2,534	1.00	1,107	0.49 (0.31, 0.77)**	1,295	0.70 (0.47, 1.04)	957	0.25 (0.12, 0.54)***	<0.001
Sex									
Female	10,378	1.00	2,571	0.65 (0.45, 0.94)*	2,952	0.69 (0.48, 0.99)*	2,472	0.54 (0.32, 0.91)*	0.003
Male	10,947	1.00	2,535	0.63 (0.47, 0.84)**	2,826	0.74 (0.56, 0.98)*	2,341	0.55 (0.37, 0.81)**	<0.001
CCI									
0	9,951	1.00	2,202	0.67 (0.47, 0.95)*	2,469	0.74 (0.53, 1.05)	2,221	0.64 (0.41, 1.00)*	0.014
1	5,622	1.00	1,348	0.62 (0.39, 0.99)*	1,586	0.83 (0.55, 1.26)	1,328	0.62 (0.35, 1.10)	0.086
2	3,213	1.00	819	0.59 (0.33, 1.04)	932	0.58 (0.33, 1.01)	731	0.37 (0.16, 0.88)*	0.005
≥3	2,539	1.00	737	0.62 (0.36, 1.08)	791	0.64 (0.36, 1.13)	533	0.26 (0.08, 0.86)*	0.007
Diabetes									
No	16,783	1.00	3,961	0.58 (0.45, 0.75)***	4,483	0.74 (0.59, 0.94)*	3,891	0.51 (0.36, 0.72)***	<0.001
Yes	4,542	1.00	1,145	0.93 (0.57, 1.52)	1,295	0.63 (0.35, 1.13)	922	0.74 (0.34, 1.59)	0.155
Dyslipidemia									
No	16,487	1.00	4,013	0.67 (0.52, 0.86)**	4,528	0.75 (0.59, 0.95)*	3,867	0.54 (0.38, 0.76)***	<0.001
Yes	4,838	1.00	1,093	0.52 (0.30, 0.90)*	1,250	0.59 (0.35, 0.99)*	946	0.54 (0.26, 1.11)***	0.014
Antihypertensives									
No (<28 days)	18,034	1.00	3,991	0.61 (0.47, 0.79)***	4,411	0.63 (0.49, 0.82)***	3,432	0.44 (0.30, 0.65)***	<0.001
Yes (≥28 days)	3,291	1.00	1,115	0.81 (0.48, 1.37)	1,367	1.15 (0.73, 1.80)	1,381	0.96 (0.55, 1.68)	0.828
Diuretics									
No (<28 days)	12,007	1.00	2,355	0.59 (0.43, 0.80)***	2,379	0.60 (0.44, 0.81)***	1,831	0.39 (0.24, 0.63)***	<0.001
Yes (≥28 days)	9,318	1.00	2,751	0.72 (0.50, 1.02)	3,399	0.91 (0.66, 1.26)	2,982	0.75 (0.49, 1.14)	0.211
Beta-blocking agents									
No (<28 days)	11,108	1.00	2,413	0.63 (0.47, 0.85)**	2,495	0.59 (0.43, 0.81)***	1,963	0.51 (0.33, 0.79)**	<0.001
Yes (≥28 days)	10,217	1.00	2,693	0.66 (0.46, 0.95)*	3,283	0.90 (0.65, 1.23)	2,850	0.58 (0.37, 0.92)*	0.041
Calcium channel blockers									
No (<28 days)	7,220	1.00	1,407	0.48 (0.32, 0.70)***	1,410	0.49 (0.33, 0.73)***	1,094	0.32 (0.17, 0.59)***	<0.001
Yes (≥28 days)	14,105	1.00	3,699	0.77 (0.58, 1.03)	4,368	0.90 (0.69, 1.18)	3,719	0.71 (0.49, 1.02)	0.080
RAAS inhibitors									
No (<28 days)	9,429	1.00	1,876	0.54 (0.39, 0.76)***	1,956	0.47 (0.32, 0.67)***	1,583	0.38 (0.23, 0.64)***	<0.001
Yes (≥28 days)	11,896	1.00	3,230	0.77 (0.56, 1.06)	3,822	1.04 (0.78, 1.38)	3,230	0.75 (0.50, 1.12)	0.370
Statins									
<28 days	16,085	1.00	3,708	0.64 (0.49, 0.82)***	4,112	0.70 (0.54, 0.89)**	3,379	0.48 (0.33, 0.69)***	<0.001
28–365 days	3,472	1.00	884	0.51 (0.26, 1.02)	969	0.90 (0.51, 1.59)	799	0.96 (0.48, 1.94)	0.748
>365 days	1,768	1.00	514	1.21 (0.47, 3.11)	697	0.89 (0.33, 2.39)	635	0.65 (0.17, 2.50)	0.572
Metformin									
<28 days	17,300	1.00	4,007	0.58 (0.45, 0.75)***	4,545	0.67 (0.53, 0.85)***	3,822	0.46 (0.33, 0.66)***	<0.001
28–365 days	1,724	1.00	417	0.44 (0.15, 1.31)	394	0.89 (0.35, 2.27)	262	1.08 (0.30, 3.86)	0.789
>365 days	2,301	1.00	682	1.99 (1.00, 3.97)	839	1.56 (0.76, 3.23)	729	1.63 (0.66, 4.00)	0.200
Aspirin									
<28 days	13,765	1.00	2,831	0.57 (0.42, 0.76)***	2,884	0.53 (0.39, 0.72)***	2,265	0.36 (0.22, 0.58)***	<0.001
28–365 days	4,511	1.00	1,254	0.75 (0.45, 1.23)	1,498	1.10 (0.71, 1.68)	1,195	0.91 (0.52, 1.62)	0.998
>365 days	3,049	1.00	1,021	0.96 (0.53, 1.74)	1,396	1.25 (0.73, 2.15)	1,353	0.90 (0.46, 1.75)	0.899

*: *p* < 0.05, **: *p* < 0.01, ***: *p* < 0.001. HR, hazard ratio.

^a^ Main model was adjusted for age, sex, CCI, diabetes, dyslipidemia, antihypertensives, diuretics, beta-blocking agents, calcium channel blockers, RAAS inhibitors, statins, metformin, aspirin, level of urbanization, and monthly income in the propensity score.

Regardless of sex, patients aged ≥65 years who had received ≥4 vaccinations had a significantly lower risk of lung cancer (aHR [95% CI] = 0.49 [0.32, 0.74] for those aged 65–74 years; aHR [95% CI] = 0.25 [0.12, 0.54] for those aged ≥75 years).

Among patients with longer durations of diuretic agent, beta-blocking agent, calcium channel blocker, RAAS inhibitor, statin, metformin, and aspirin use, there was no significant difference in lung cancer occurrence between the vaccinated and unvaccinated groups, even after 4 influenza vaccinations (aHR [95% CI] = 0.75 [0.49, 1.14] for diuretics ≥28 days; aHR [95% CI] = 0.71 [0.49, 1.12] for RAAS inhibitors ≥28 days; aHR [95% CI] = 0.65 [0.17, 2.50] for statins ≥365 days; aHR [95% CI] = 1.63 [0.66, 4.00] for metformin ≥365 days; aHR [95% CI] = 0.90 [0.46, 1.75] for aspirin ≥365 days).

## Discussion

In Taiwan, in 2007, the nationwide prevalence of hypertension was 25% in men and 18% in women, increasing to 47% in individuals aged ≥60 years [30]. Community-based data on a 10 years follow-up cohort in Taiwan revealed an increased incidence among individuals with prehypertension, obesity and metabolic syndrome [31]. Recent clinical studies have reported that hypertension alone, as well as diuretic and antihypertensive medications, are strongly associated with various types of cancers [9–12, 32]. Hypertension together with metabolic syndrome also increases the risk of cancer through the stimulation of various inflammatory, metabolic, and hormonal signaling pathways [33].

Lung cancer is the second most common cancer in Taiwan regardless of sex [34]. In a recent study, the incidence of lung adenocarcinoma increased, and that of lung squamous cell carcinoma decreased in Taiwan [35]. The risk factors for lung cancer in Taiwan have been reported differently for different types of lung cancer. Occupational exposure to asbestos and working in kitchens were associated with lung adenocarcinoma. Smoking/passive smoking exposure, tuberculosis history, chronic bronchitis and occupational exposure to asbestos were associated with lung squamous cell carcinoma [36]. In a recent study that investigated lung cancer survival in Taiwan, the 5 years overall survival rate among all lung cancer patients was only 25.0% [37]. Therefore, the primary prevention of lung cancer in the Taiwanese population is an important target of study.

In this cohort study, the patients with hypertension in the vaccinated group tended to be older and female compared with those in the unvaccinated group. Significantly more patients in the vaccinated group had taken antihypertensive medications, including diuretics, beta-blocking agents, calcium channel blockers, and RAAS inhibitors, and had used medications such as statins, metformin, and oral aspirin for a prolonged period. This finding indicates that, relative to those in the unvaccinated group, more patients in the vaccinated group had metabolic disorders such as hyperlipidemia and DM and were receiving treatment. The incidence of lung cancer in the vaccinated group was significantly lower than that in the unvaccinated group (aHR = 0.56, 95% CI = 0.47–0.67). To our knowledge, this is the first population-based cohort study to reveal that influenza vaccination significantly reduced the risk of lung cancer among patients with hypertension.

The mechanisms linking metabolic syndrome and cancer risk are not fully understood. Metabolic syndrome may be a surrogate marker for other cancer risk factors, such as decreased physical activity, consumption of calorie-dense foods, high dietary fat intake, low fiber intake, and oxidative stress. Excess adiposity, particularly visceral obesity, results in a state of chronic systemic low-grade inflammation, attributed to the production of inflammatory cytokines by both adipocytes and infiltrating immune cells, creating a protumorigenic environment [38]. Although the relationship between hypertension and cancer pathogenesis is unclear, an animal study revealed that RAAS activation in rats with hypertension plays a role in the early stages of colorectal carcinogenesis by inducing oxidative stress and chronic inflammation [39]. Studies have also reported that numerous pathophysiologic pathway abnormalities, such as programmed cell death, VEGFs, HIFs, RAAS, IGF formation, tumor angiogenesis other than induced chronic inflammation, and oxidative stress, are involved in hypertension-related cancer risk [12, 40]. These disorders share the complex mechanisms of tumorigenesis. In addition, antihypertensive medications, especially angiotensin-converting enzyme inhibitors, are associated with the risk of lung cancer. Seasonal influenza vaccination generates systemic CD8^+^ T-cell-mediated antitumor immunity [41], which also boosts the response to antitumor treatment.

In a previous study conducted in Taiwan, the risk of lung cancer significantly increased after influenza virus infection [42]. Moreover, with increasing total cumulative exposure to influenza, the risk of lung cancer occurrence also increased [42]. Among patients with lung cancer, the risk of disease progression increased after influenza virus infection [43]. Possible mechanisms previously reported include changes in the tumor microenvironment and a weakening of the effectiveness of antitumoral treatments [43]. It has been reported that after influenza virus infection, significantly increased levels of active viral RNA remnants persist up to 26 weeks post-infection and may potentially be one of the mechanisms of chronic lung disease occurrence [44]. Therefore, if influenza viral infection could be prevented even by a single dose of the influenza vaccine, the risk of chronic lung disease development could be reduced, and the future risk of lung cancer would also potentially decrease.

This population-based cohort study revealed that annual influenza vaccination significantly reduced the risk of lung cancer among patients with hypertension, demonstrating that a higher number of vaccinations was related to a stronger protective effect. Subgroup analyses were performed according to vaccination status, and we evaluated the dose-dependent effects on lung cancer events. Of the 37,022 eligible patients with hypertension, 15,697 (42.4%) had received an influenza vaccination, and 21,325 (57.6%) had not.

The dose-dependent protective effect of the influenza vaccination for all ages and both sexes (significantly for individuals aged >75 years) was also identified in our other studies [24, 25]. Influenza vaccination-related immunomodulation might explain this phenomenon. Because this was an observational study, no data were available on other possible precancerous indicators related to oncogenesis, as indicated by immunity-related cells such as T helper cells, Toll-like receptors, and B cells [45–47]. Previously, influenza vaccination was revealed to have an augmenting effect on natural killer cell (NK) activity; after the first influenza vaccination, older patients exhibited a high level of postvaccination NK activity and associated higher anti-hemagglutinin antibodies, leading to a lower incidence of respiratory tract infections. After the second vaccination, most older patients with chronic medical conditions and high levels of NK cell activity, who had not attained protective levels of anti-hemagglutinin antibodies after the first vaccination, developed this protection [48]. This finding indicated that influenza vaccination-induced NK cell activity in older people stimulates a protective humoral anti-hemagglutinin response, resulting in protection against respiratory tract infections. The role of NK cell function in tumor suppression was also identified [49]. Similarly, some studies have indicated that peripheral NK cells are able to robustly trigger increased recall interferon-gamma responses for 6 months after influenza vaccination [50]. Although studies in recent years have identified multiple suppressive loops to NK cell function in the tumor microenvironments, the mechanism underlying humoral and immune cells and cytokines, including the interferon response in tumor microenvironments, requires further study. The role of blood pressure regulation and changes in tumor environments as well as the immunomodulatory role of influenza vaccination under different clinical conditions also merit further clinical studies.

However, in the present study, the potential chemopreventive effect of influenza vaccination was not observed in patients with a longer duration of antihypertensive and other metabolic medication use. With a longer duration of medication usage, it is possible that these patients had more severe or irreversible causes of these comorbidities. In previous studies, cardiovascular disease, diabetes and dyslipidemia have been reported to be associated with a higher cancer risk [51–53]. Therefore, the potential chemoprotective effect of influenza vaccination might be countered. Although a significantly lower risk of lung cancer was observed among patients with a higher CCI after influenza vaccination in the present study, future studies among patients with different clinical scenarios are warranted to validate the results of the present study.

Several limitations to this study, similar to those in other observational studies based on clinical databases, must be considered. First, data on smoking, a key environmental risk factor for lung cancer and a cardiovascular risk factor, are not available in the NHIRD. In Taiwan, the smoking rates for men and women aged >55 years were 20.5% and 2.4% in 2002 and 14% and 1.53% in 2012, respectively [53]. The lower smoking rate among women might be related to traditional Chinese cultural factors. Tobacco use has been reported to be the main cause of lung cancers, and the risk of developing lung cancer is 20–40 times higher among smokers than among non-smokers [54]. Because it damages the local cellular and humoral immunity in the respiratory system [55, 56], smoking facilitates infection with the influenza virus and subsequent pneumonia [57, 58]. A study also revealed that smokers and ex-smokers had an increased risk of influenza-related hospitalization [59]. Second, hypertension, comorbidities and medication use were identified according to ICD-9-CM codes or drug codes alone. We used propensity score matching to reduce the selection bias by accounting for the covariates through a logistic regression mode [28]. We further performed additional subgroup analyses and made adjustments for confounding factors, using several stratifications for the comparison between groups to avoid the influence of interference factors. Third, an intrinsic weakness of the NHIRD is the lack of biochemical data; thus, we were unable to assess the duration or severity of hypertension and other comorbidities in our study population. We investigated the short- and long-term duration of medication use and CCI, which might partially reflect the possible severity of each comorbidity under these medical treatments. Data on other unmeasured confounding factors, including body mass index, alcohol intake, and use of over-the-counter medications, were not available for our study population from the database. However, in Taiwan, patients over 55 years with hypertension are eligible for publicly funded influenza vaccinations. We therefore believe that this factor is unlikely to have influenced our results. In addition, all patients included in this study were from an Asian population, and ethnic susceptibility is unclear. Therefore, our results should be cautiously extrapolated to non-Asian populations. However, considering the magnitude and significance of the observed effects, these limitations are unlikely to have influenced the results. Fourth, the present study analyzed the data between 2011 and 2012. New diagnostic tools, such as low-dose computed tomography, could possibly increase the incidence of lung cancer. Future studies that investigate data from more recent times are warranted. Finally, because the present study was not a prospective randomized blinded study, a cause–effect relationship could not be established. This type of study is needed to verify our findings.
